# Characteristics and Etiologies of Chronic Scrotal Pain: A Common but Poorly Understood Condition

**DOI:** 10.1155/2017/3829168

**Published:** 2017-03-02

**Authors:** Aosama Aljumaily, Hind Al-Khazraji, Allan Gordon, Susan Lau, Keith A. Jarvi

**Affiliations:** ^1^Division of Urology, Department of Surgery, Mount Sinai Hospital, University of Toronto, Toronto, ON, Canada; ^2^Murray Koffler Urologic Wellness Centre, Mount Sinai Hospital, Toronto, ON, Canada; ^3^Wasser Pain Management Centre, Mount Sinai Hospital, Toronto, ON, Canada; ^4^Lunenfeld-Tanenbaum Research Institute, Mount Sinai Hospital, Toronto, ON, Canada; ^5^Institute of Medical Sciences, University of Toronto, Toronto, ON, Canada

## Abstract

Chronic scrotal pain (CSP) is a common and debilitating condition, but the underlying characteristics and etiology of CSP are poorly understood. The objective of this study is to identify the characteristic and etiologies of CSP. Men presenting for management of CSP completed a standardized questionnaire and underwent a complete physical examination. From Feb 2014 to Sep 2015, a total of 131 men (mean age 43) with CSP were studied. The CSP was of long duration (mean of 4.7 ± 5.95 years) and dramatically affected men's lives, with adverse effects on normal activities (71.%), ability to work (51.90%), and sexual functioning (61.8%). 50.4% felt depressed on most days, and 67.17% felt either unhappy or terrible with their present condition. Physical examination revealed that the epididymis was the most common tender area found in 70/131 men (53.43%), though a musculoskeletal source for the pain was found in 9.9%. Neuropathic changes were found in 30%. For close to half of the men (43.5%) we were unable to identify any potential cause for the CSP. This study characterizes the dramatic impact that CSP has on the lives of men, while providing an understanding of the common etiologies.

## 1. Introduction

While chronic scrotal pain (CSP), defined as pain in the scrotum of more than 3-month duration, appears to be a very common condition, there are very few studies on the actual incidence of CSP [1]. Ciftci et al. reported that 4.75% of all men presenting to urology clinics for other reasons had CSP [2]. A similar incidence was found in our centre, where Forbes et al. found that 4.3% of all men presenting to a dedicated male infertility clinic self-identified as having CSP (unpublished data). A lower incidence was reported in Switzerland of 350 to 400 cases of CSP per 100,000 men annually [3]. These estimates were based on survey of urologists' recollections of the numbers of men with CSP they treated and as such will be an underestimate of the true incidence [3].

For a condition which is this common, there is remarkably little known or published about the causes or the characteristics of CSP [4]. Interestingly, there are no guidelines specifically for the investigation or management of CSP, though the European Urology Association included CSP in the guidelines for the management of pelvic pain, but with a very limited section on the guidelines for management of CSP [5].

We have a number of reports on the different potential causes of CSP but no information of the frequency of each of these potential etiologies [2, 4, 5]. In general, CSP could be due to pain arising from the scrotal contents directly or referred from the abdomen/inguinal region, the retroperitoneum, or the nervous system [6]. While there are several papers listing the causes of CSP, there are no reports on the frequencies of any of the causes of CSP [6]. Often no etiology for the CSP is identified [1, 4, 5].

Diagnosing a potential etiology for CSP is also complicated by neuropathic changes which may occur in patients with chronic pain. While the initiating event may be a vasectomy, infection, or other conditions, nervous system plasticity is thought to result in upregulation of both central and peripheral neuropathic pathways in response to chronic pain, leading to neuropathic components to the chronic pain [7]. These neuropathic changes may remain even if the initiating event has disappeared. The neuropathic changes may also be bilateral. A source of pain on one side may also lead to chronic pain on the contralateral side: nerves from the pelvic plexus cross over to the contralateral pelvic plexus, which may play a role in creating a contralateral effect in the presence of unilateral pathology (e.g., varicocele) [8].

While neuropathic changes associated with CSP are recognized, the frequency of neuropathic changes has not been reported to date.

There is also little published information on the clinical condition of CSP, including the characteristics of the pain, the factors which modify the pain (exacerbating and relieving factors), and the impact of the CSP on men's quality of life, ability to work, and ability to have normal social lives [5].

With limited published information on the etiology and characteristics of CSP, it is not surprising that the optimum method for the evaluation and treatment of this syndrome remains uncertain. The objective of this study is to survey a patient population with CSP to identify the different types of etiologies of CSP, the frequency of the different etiologies, and the different types of characteristics of the CSP in men.

## 2. Material and Methods

This assessment was performed at the Multidisciplinary Orchialgia Clinic (MOC) located at Mount Sinai Hospital in Toronto, Canada, in which the patient is evaluated simultaneously by both urologists with special expertise in CSP and neurologists specialized in chronic pain conditions.

Mount Sinai Hospital local ethical committee approval was obtained prior to commencement of the study and the patients in this manuscript have given written informed consent to publication of their case details. This is a retrospective review of a prospectively collected database of men presenting to a university program specializing in chronic scrotal pain.

The men completed a standardized questionnaire to elicit information on the pain severity, pain duration, potential etiology, quality, location, progression, previous treatments, and impact of different activities on severity of CSP (see Supplementary Material available online at https://doi.org/10.1155/2017/3829168 for full questionnaire). The information on the questionnaires was retrospectively reviewed.

The severity of patients' average and most severe episodes of pain was recorded in the questionnaire based on the standardized Numeric Rating Scale (NRS) for pain from 0 to 10. The NRS is well known and widely accepted as a valid measure of pain severity in adults [1]. We also developed internally an additional nonvalidated question to determine the frequency of severe pain experienced by the men.

The impact of CSP on men's sexual and work activities was graded as none, only a little, some, or a lot using the published questionnaire from Nickel et al. [9].

In addition, we included standardized quality of life and depression questions in the questionnaire [9] and depression score questions [10] as well as standardized questionnaires to identify symptoms of androgen insufficiency (ADAM score) [11].

There were no existing questionnaires that we found on CSP potential etiologies, location, and characteristics or modifying factors so we developed but have not validated this portion of the questionnaire internally. Other parts of the questionnaire included general information on demographics, general health and lifestyle factors, and questions on previous therapies for the CSP.

Physical examination was used to identify any palpable abnormalities of the scrotal contents, inguinal region, or the local musculoskeletal structures (adductor tendons, conjoint tendon, etc.). Tender areas were identified by gentle palpation of the above structures. Often more than one area of tenderness was identified and if so we recorded this information taking care to identify the degree of tenderness in each area (e.g., head of left epididymis is more tender than tail of left epididymis). A focussed neurological examination including sensory testing was performed to identify neuropathic changes in the lower abdomen, groin, and legs.

Imaging was not routinely performed on our patients but was reserved for those we suspected had an abnormality in the testis (scrotal ultrasound) or those who had no tenderness found in the scrotum, inguinal region, or the groin (abdominal imaging to identify a retroperitoneal or renal cause for the CSP).

The results were analyzed with descriptive statistics.

## 3. Results

From Feb 2014 to Sep 2015, a total of 131 men presenting for assessment of CSP completed questionnaires. The mean age of the men was 43 ± 12 (SD) years with a mean duration of CSP of 4.7 ± 5.95 years.

There were a variety of potential causes for the CSP reported by the patients including the following:

1. Unknown: 43.5% (Table 1)
2. Previous vasectomy: 20.6%
3. Testicular trauma: 12.2%
4. Documented testicular, prostate, or epididymal infection: 11.5%
5. Hernia repair: 4.6%
6. Other reported potential causes (varicocelectomy, TURP, hydrocelectomy, donor nephrectomy, orchidectomy, and knee surgery: each was related definitively to the surgery by the patient)

The men often complained of other existing chronic pain conditions such as chronic bowel pain found in 28.24%, migraines in 20%, and fibromyalgia in 6.9%. While the pain was described as being in the testes by most of the patients, the most common area of tenderness identified by careful physical examination was the epididymis in 53.43%. Tenderness was found in the testicle in 25.19%, the site of vasectomy in 11.5%, and the conjoint tendon in 10%.

Neuropathic changes were found in the groin regions in close to 30% of the men: there was evidence of hypersensitivity with increased sensitivity to light touch found in 8.4% while conversely decreased sensation to light touch was noted in 5.34% of men. Hyperalgesia was also noted with increased sensitivity to pin prick found in 11% of patients and decreased in 5.3%.

**Figure 1:**
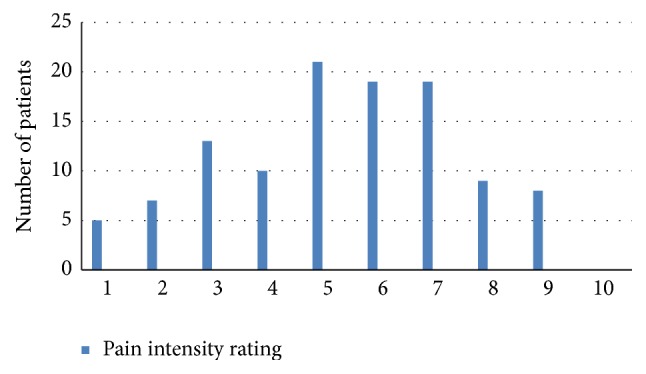
Average pain score in patients with CSP.

**Figure 2:**
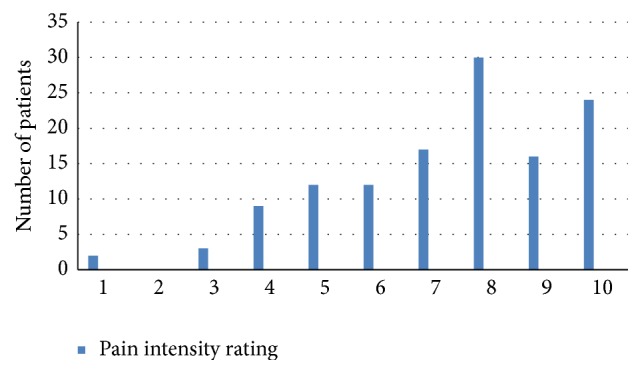
Pain levels with episodes of severe exacerbations.

**Figure 3:**
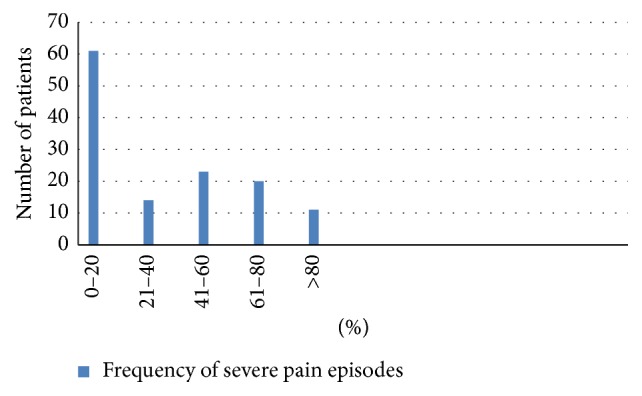
Frequency of severe pain episodes in patients with CSP.

For most men, the scrotal pain was quite severe, but for almost all men the pain tended to wax and wane with time. On average, severe pain episodes (mean pain severity of 7.2 ± 2 on a 10-point numeric pain scale) affected men 40% ± 30% of the time. The level of pain severity was similar in the groups of men with different etiologies for the scrotal pain. Most men had some constant background pain, which the men rated as a pain level of 5.7 ± 2.3 (Figures 1–3). The quality of the pain was also extremely variable, with the pain described as sharp in 52.7%, dull in 38.2%, burning in 6.9%, and throbbing in 2.3%.

The CSP was exacerbated by a number of factors such as sitting for 59.5% of the men, movement (48.1%), tight clothing (44.3%), ejaculation (36.6%), and sex (35.9%). On the other hand, certain factors improved the pain such as lying down (48.85%), sitting (24.4%), and hot baths (4.6%). Unfortunately, for the vast majority of the men, the pain was either becoming more severe with time (49.6%) or continuing at the same pain level (33.6%).

The impact of the CSP on the lives of men was very significant, with 93/131 (71%) of the men noting that the CSP symptoms prevented them from doing normal social activities, 51.9% found the CSP interfered with their ability to work, and 61.8% noted a negative impact on their sexual function and enjoyment. The severity of the pain and the impact on the men's lives had an effect on the men's mood, with 50.4% of the patients who answered the question saying they felt depressed on most days. Many more felt either unhappy or terrible (67.2%) with their present condition.

In general, the men had often tried other therapies to manage their pain. Before presenting to our clinic, they

1. used over the counter medications: 60.30%,
2. had been prescribed antibiotics: 58.8%,
3. used anti-inflammatory medications: 65.6%,
4. had used neuropathic pain medications: 31.3%.

The ongoing use of narcotics to manage the pain was extremely common (35.9%), while 38.2% of the men used antidepressants. Only 2 patients (1.52%) used methadone and another 2 used nabilone. Medical marijuana use was uncommon in this group (2.29%).

### 3.1. Discussion

CSP remains a challenge to clinicians and patients alike. Clinicians are often faced with a patient with a debilitating, high impact and chronic condition. The pain in our patients was severe, usually progressive, was worsened by even simple daily activity, and limited many of the activities of daily life like work, social activities, and sports. Many of the men were depressed and most felt that their condition was “terrible.” A similar finding was published by Nickel et al. [9].

While chronic pain of any origin may be debilitating, limit activities, and lead to poor quality of life and depression, there are some characteristics that are much more common in men with CSP: sex and ejaculation commonly exacerbate the pain for men with CSP, with the men describing impaired sexual function and enjoyment. In addition, sitting for the majority of men exacerbated the pain, while lying down improved the pain levels.

While there is very little published information on CSP, it appears to be an extremely common condition [12, 13], affecting potentially more than 4% of men [2, 12, 13]. This lack of publications and information on CSP makes the challenge to clinicians even greater as there is a lack of information on the frequency of the different potential causes of CSP, the strategies to investigate and manage men with CSP, and outcomes of therapies.

This study and others have identified multiple potential causes for the CSP, varying from previous surgery (most notably vasectomies), infections, trauma, referred pains, and medications. Possibly not surprisingly, for almost half of the men in this series, no potential cause for the CSP was identified.

The use of the term “potential causes” must be emphasized, since there is often no clear direct link between these “potential causes” and the CSP: for example, men may have had a vasectomy (which is common) and also have CSP (also common).

This study does emphasize the variability of presentation, characteristics, and etiology of CSP. It should also be recognized that scrotal pain is not a synonym for scrotal pathology and other sources of referred pain should be evaluated. Many of our patients presented with what by history was chronic scrotal pain, but the source of the pain was not from the scrotal contents. Musculoskeletal pain was the source of the CSP in close to 10% of the men. In addition, neuropathic changes occur in 30% of men with CSP. What may have begun as nociceptive pain arising from the scrotal contents has developed into neuropathic pain and neuroplasticity modified the central and peripheral nerves leading to hyperalgesia and allodynia.

To further complicate matters, men with CSP have significant limitations on their normal activities and often expressed feelings of depression.

For a condition with this type of variability of presentation, characteristics, and etiology, one would also expect a variability of response to activities and therapies. Most patients reported that lying down helped reduce the scrotal pain, while most found that sex/ejaculation and tight clothing exacerbated the pain. Interesting, again illustrating the variability of CSP, a similar number of men found that sitting improved or exacerbated the pain. Patients had used a variety of different medications (often multiple different medications for the same patient) with different responses. The variability of response to medical therapies should not be surprising given the variety of etiologies of CSP and the very high frequency (43.5%) of an unknown etiology.

Clearly, evaluating patients with chronic scrotal pain should be comprehensive and must include evaluations to rule out medically important and treatable urological causes including tumours, intermittent torsion, infection, and varicocele. It is also important to remember that a significant fraction of the men presenting with CSP symptoms have a musculoskeletal or a neuropathic source for the symptoms.

While the history may help to differentiate chronic scrotal contents (testis, epididymis, and paratesticular structures) pain from musculoskeletal or neuropathic pain, a thorough physical examination is essential, providing information about the source of the pain in most cases [14]. This will help determine if the scrotal contents are tender and are the source of the CSP. We also recommend careful examination of the inguinal canal and the adductor tendon. Quite often (just under 10% in our series) the pain is found to arise from a MSK source (the conjoint tendon or the adductor tendon). Additionally, we suggest a focussed neurological examination of the groin to identify increased or decreased sensation to light touch and pin prick. Close to 30% of the men had a neuropathic component of the pain diagnosed by a focussed neurological examination.

Finally, it is also important to provide a psychosocial evaluation in order to determine whether there is any disability associated with the pain and if there are signs or symptoms of depression. Similar to our study, it has also been reported in the literature that a significant number of patients who suffer from chronic orchialgia express signs of major depression and a significant number of these patients have clinical dependencies [15].

While CSP is a challenging condition to investigate and manage, a thorough history to identify the causes and impact of CSP on the patients coupled with a physical examination of the scrotum and groin often identifies the cause and the origin of the pain. While management is often multidisciplinary (pain medicine specialists, neurologists, psychiatrists/psychologists, physiotherapists, orthopaedics, sports medicine, general surgery, and urologists), an understanding of the cause, characteristics, and origin of the CSP is essential to tailor consultations to the appropriate services.

### 3.2. Limitations

This is a single centre study so the results may not be generalizable to the types of men with CSP seen at other pain centres. In addition, the men who present for investigation and therapy for the CSP are likely to be more severely affected than a general population of men with CSP, which biases our sample to the more severely affected men with CSP.

## 4. Conclusion

CSP is an extremely variable condition with a wide variety of etiologies, effects on quality of life, exacerbating factors, and treatment responses. This condition represents a challenge to patients and clinicians. A multidisciplinary approach is needed to optimally manage the patients with CSP.

## Supplementary Material

This standardized questionnaire to obtain detailed information about the patient's chronic scrotal pain, previous therapies for the pain, general health and previous health issues and therapies, depressive symptoms and sexual health is completed by the patients prior to the first consultation.

## Figures and Tables

**Table 1:** Identified causes of the CSP.

Cause of the CSP	Number of patients
Vasectomy	27 (20.61%)
Trauma	16 (12.21%)
Infection	15 (11.45%)
Hernia repair	6 (4.58%)
Epididymal cyst	2 (1.52%)
Other identified causes	8 (6.10%)
Unknown	57 (43.51%)
