# Supplementary material for: Characteristics and Etiologies of Chronic Scrotal Pain: A Common but Poorly Understood Condition
Source: Pain Res Manag. 2017 Mar 2;2017:3829168. doi: 10.1155/2017/3829168 (PMC5352901; doi:10.1155/2017/3829168)
Supplement: Supplementary file 1 — This standardized questionnaire to obtain detailed information about the patient's chronic scrotal pain, previous therapies for the pain, general health and previous health issues and therapies, depressive symptoms and sexual health is completed by the patients prior to the first consultation. [file 3829168.f1.pdf]

# The Murray Koffler Urologic Wellness Centre

**60 Murray Street, 6th floor, Toronto, ON, M5T 3L9**

*You have been referred to the Multi-Disciplinary Scrotal Pain Clinic. We would ask that you please fill in the following questionnaire as completely and accurately as possible before you see the doctor. All provided information will remain confidential. It will be released only with your written permission.*

Today's Date: ____/____/______ (DD/MM/YYYY)    MSID#: _______________

Name: _______________, _______________ (Last Name) (First Name)    DOB: ____/____/______ (DD/MM/YYYY)

Referring MD: _________________________

Occupation: ________________________    Number of years at current job: _______

Previous occupation: _________________

Do you have any allergic reactions to medications? Y/N

Which medications and what type of reaction? ____________________________

**Pain/Discomfort Location and history:**

When did your scrotal pain/discomfort begin?__________ (year)

Was there something which started the pain? Y/N

Vasectomy [ ]    Hernia repair [ ]    Injury [ ]    Infection [ ]

Other______________________________________________

Since the pain began has the pain/discomfort generally become

better [ ], same [ ] or worse [ ]

What can you do to improve the pain?

Standing [ ], Lying [ ], other _________________________________________

What makes the pain worse?

Tight clothing [ ], Sitting [ ], Movement [ ], Sex [ ], Ejaculation [ ]

Other_______________________

How would you describe the pain/discomfort?

Sharp [ ], Shooting [ ], Pressure [ ], Dull [ ], Achy [ ], Electric [ ], Numb/Tingling [ ]

Other_______________________________________________

**Areas of pain/discomfort:**

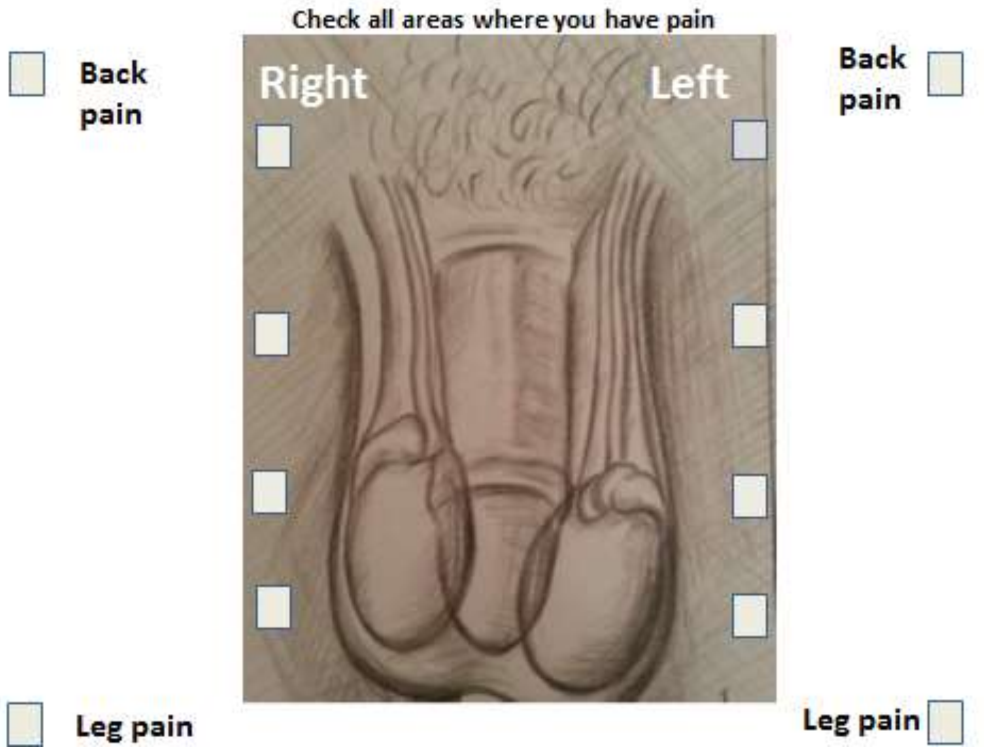


**Present Pain levels and frequency of pain:** *(circle the most accurate answer)*

| | | | | |
|---|---|---|---|---|
| **When you have pain, how bad was your AVERAGE pain in the last month?** | **1 2 3 4 5 6 7 8 9 10**<br>No pain … Pain as bad as you can imagine | | | |
| **How bad was your MOST SEVERE (WORST) pain in last month?** | **1 2 3 4 5 6 7 8 9 10**<br>No pain … Pain as bad as you can imagine | | | |
| What percentage of the time did you have the MOST SEVERE pain in the past month? (YOUR BEST GUESS) | **10% 20% 40% 60% 80% 100%** | | | |
| How much have your symptoms kept you from doing the **kinds of things you would usually do**, over the last month? | None | Only a little | Some | A lot |
| How much have your symptoms kept you from **performing your normal work**? | None | Only a little | Some | A lot |
| How much have your symptoms kept you from **normal sexual activity?** | None | Only a little | Some | A lot |
| How much did you **think about your symptoms**, over the last month? | None | Only a little | Some | A lot |

Describe the impact of the scrotal pain on your life

______________________________________________________________

Describe what changes you have made in your life because of the scrotal pain

______________________________________________________________________________

______________________________________________________________________________

If you were to spend the rest of your life with your symptoms just the way they have been during the last month, how would you feel about that?

1 Delighted
2 Pleased
3 Mostly satisfied
4 Mixed (about equally satisfied and dissatisfied)
5 Mostly dissatisfied
6 Unhappy
7 Terrible

**<u>What therapies have been used to treat the scrotal pain/discomfort?</u>**

**What medications have been used to treat the scrotal pain/discomfort?**

[ ] Over the counter pain medications like Tylenol, Ibuprofen, Motrin
[ ] Prescribed pain medications like codeine, oxycodone, fentanyl, morphine
[ ] Antibiotics
[ ] Anti-inflammatories like ibuprofen, celebrex, vioxx
[ ] Medications for nerve pain like lyrica, gabapentin, carbamazepine
[ ] Anti-depressants like elavil, tofranil, imipramine
[ ] Other________________________________________________________________

**Surgery/procedures to treat scrotal pain/discomfort**

| | | |
|---|---|---|
| Vasovasostomy (Vasectomy Reversal) | L/R/Both Sides/No | Date: _______ |
| Varicocelectomy | L/R/Both Sides/No | Date: _______ |
| Removal of Epididymis | L/R/Both Sides/No | Date: _______ |
| Removal of Testicle | L/R/Both Sides/No | Date: _______ |
| Nerve blocks | L/R/Both Sides/No | Date: _______ |
| Removal of Nerves to the testicle | L/R/Both Sides/No | Date: _______ |

Other ___________________________/_____________________________

**<u>Medical History</u>**

Height _____Feet______inches Weight___________ Lbs

Diabetic Y/N

**Do you have any other chronic pain issues? Y/N**

Bowel/stomach Y/N Fibromyalgia Y/N Headaches/Migraines Y/N

**Infections** *(Please circle Y or N)*

| | | | | | | | |
|---|---|---|---|---|---|---|---|
| Gonorrhea | Y/N | Genital warts | Y/N | Chlamydia | Y/N | Syphilis | Y/N |
| Herpes | Y/N | | | | | | |
| Bladder | Y/N | Prostate | Y/N | Testis | Y/N | Penis | Y/N |
| SHINGLES | Y/N | | | | | | |

Other ____________________________________________________

**Past Surgical History** *(Please circle Y or N, and fill in the appropriate blanks if Yes)*

| | | |
|---|---|---|
| Hernia: | L/R/Both Sides/No | Date: _______/_______ |
| Undescended Testes | L/R/Both Sides/No | Date: _______/_______ |
| Vasectomy | Yes / No | Date: _______ |
| Abdominal/Pelvic Surgery | Yes / No | Date: _______ |

Other ___________________________/_____________________________

**Lifestyle**

| | | | |
|---|---|---|---|
| Smoking | | Y/N | <1 / 1 / 2 / ≥2 pack(s) per day |
| Alcohol | | Y/N | <1 / 1 / 2 / 3 / ≥4 drinks/per day |
| Marijuana | Used in last six months? | Y/N | |
| Cocaine | Used in last six months? | Y/N | |
| Other drugs | Used in last six months? | Y/N | Type ____________________ |

**Androgen (Male Hormone) Deficiency and Sexual Dysfunction Questionnaire**

1. Do you have a decrease in libido (sex drive)? Yes No
2. Do you have a lack of energy? Yes No
3. Do you have a decrease in strength and/or endurance? Yes No
4. Have you lost height? Yes No
5. Have you noticed a decreased enjoyment of life? Yes No
6. Are you sad and/or grumpy? Yes No
7. Are your erections less strong? Yes No
8. Have you noted a recent deterioration in your ability to play sports? Yes No
9. Are you falling asleep after dinner? Yes No
10. Has there been a recent deterioration in your work performance? Yes No

Circle the most correct answer

| | | | | |
|---|---|---|---|---|
| During the past month, have you **often** been bothered by feeling down, depressed, or hopeless? | Not at all | Several days | More than half the days | Nearly every day |
| During the past month, have you **often** been bothered by little interest or pleasure in doing things? | Not at all | Several days | More than half the days | Nearly every day |
